# New Insights Into Peptide Cannabinoids: Structure, Biosynthesis and Signaling

**DOI:** 10.3389/fphar.2020.596572

**Published:** 2020-12-09

**Authors:** Agustín Riquelme-Sandoval, Caio O. de Sá-Ferreira, Leo M. Miyakoshi, Cecilia Hedin-Pereira

**Affiliations:** ^1^ Laboratory of Cellular Neuroanatomy, Institute of Biomedical Sciences, Federal University of Rio de Janeiro, Rio de Janeiro, Brazil; ^2^ Institute of Biophysics Carlos Chagas Filho, Federal University of Rio de Janeiro, Rio de Janeiro, Brazil; ^3^ VPPCB-Fiocruz, Rio de Janeiro, Brazil; ^4^ National Institute of Science and Technology in Neuroimmunomodulation (INCT-NIM), Rio de Janeiro, Brazil

**Keywords:** endocannabinoid system, cannabinoid receptors, cannabinoid biosynthesis, hemopressin peptides, cannabinoid signaling

## Abstract

Classically, the endocannabinoid system (ECS) consists of endogenous lipids, of which the best known are anandamide (AEA) and 2 arachidonoylglycerol (2-AG), their enzyme machinery for synthesis and degradation and their specific receptors, cannabinoid receptor one (CB1) and cannabinoid receptor two (CB2). However, endocannabinoids also bind to other groups of receptors. Furthermore, another group of lipids are considered to be endocannabinoids, such as the fatty acid ethanolamides, the fatty acid primary amides and the monoacylglycerol related molecules. Recently, it has been shown that the hemopressin peptide family, derived from α and β chains of hemoglobins, is a new family of cannabinoids. Some studies indicate that hemopressin peptides are expressed in the central nervous system and peripheral tissues and act as ligands of these receptors, thus suggesting that they play a physiological role. In this review, we examine new evidence on lipid endocannabinoids, cannabinoid receptors and the modulation of their signaling pathways. We focus our discussion on the current knowledge of the pharmacological effects, the biosynthesis of the peptide cannabinoids and the new insights on the activation and modulation of cannabinoid receptors by these peptides. The novel peptide compounds derived from hemoglobin chains and their non-classical activation of cannabinoid receptors are only starting to be uncovered. It will be exciting to follow the ensuing discoveries, not only in reference to what is already known of the classical lipid endocannabinoids revealing more complex aspects of endocannabinoid system, but also as to its possibilities as a future therapeutic tool.

## Introduction

The discovery of the nature of the endocannabinoid system (ECS) and the ever-growing information about novel receptors and ligands, have progressively revealed the complexity of the ECS making it worth revisiting. The ECS, which consists of endogenous ligands referred as endocannabinoids (eCBs), specific receptors and the pathways for synthesis and degradation of cannabinoid compounds, was first characterized in the central nervous system (CNS). More recently, it has been recognized as a key mediator of several aspects of human physiology being present in the entire body. Nowadays, ECS is known to be present almost everywhere in the human body and it functions by maintaining its homeostasis (Alger, 2013). To maintain the body’s balance, structural and functional integrity, the ECS safeguards the biochemical balance by controlling fundamental functions on the whole body (Zou and Kumar 2008; Maccarrone et al., 2014, Maccarrone, 2017). Until recently, endogenous or phytocompounds acting on the ECS were all thought to be of lipid nature. However, it was recently found that cannabinoid receptor activity can be modulated by hemoglobin-related peptides (Heimann et al., 2007; Gomes et al., 2009). To what is already known of the classical lipid eCBs, the novel peptide compounds derived from hemoglobin chains, which comprise non-canonical cannabinoid signaling pathways for controlling these events, are only starting to be described.

The hemopressin peptides are a family of endogenous polypeptides that act as modulators of the cannabinoid receptors, which have different lengths, ranging from 12 to 23 amino acids (Bauer et al., 2012). There are N-terminal extended forms of hemopressin (Hp), the first peptide to be discovered (Rioli et al., 2003; Dale et al., 2005). Hp is a 9-residue amino acid peptide (PVNFKLLSH) derived from the hemoglobin α subunit. Hp is not an endogenous peptide, but rather an extraction artifact that acts as antagonist/inverse agonist of cannabinoid receptor 1 (CB1) (Heiman et al., 2007; Gomes et al., 2009). Growing evidence suggests that N-extended hemopressin peptide is in fact produced in brain and in other organs (Gomes et al., 2009; Bauer et al., 2012; Gelman et al., 2013; Petrucci et al., 2017). Moreover, RVD hemopressin (RVD-Hp), a 12-residue amino acid peptide, was considered the shortest and most abundant of these peptides in brain (Bauer et al., 2012; Gelman et al., 2013). RVD-Hp was first characterized as a CB1 receptor agonist and later as a CB1 negative allosteric modulator and a positive cannabinoid receptor 2 (CB2) allosteric modulator (Bauer et al., 2012; Petrucci et al., 2017).

Besides, there are observations supporting the hypothesis that hemopressin peptides activate signaling pathways distinct from classical cannabinoids. Also, these peptides directly or indirectly modulate the functions of other receptors beyond CB1 and CB2 and also interact with other components of the ECS and other endogenous systems in the regulation of diverse physiological functions. These findings reveal a novel endogenous component in the ECS that, due to its specific protein composition, provides new pharmacological and biological properties on cannabinoid signaling. However, more studies are needed in order to elucidate the biosynthesis and the mechanisms by which hemopressin peptides modulate cannabinoid receptors and other components of the ECS.

We believe that hemopressin peptides might be an important tool for dissecting cannabinoid functions, contributing with other regulatory roles, as we will see ahead. In this review, we briefly describe the current knowledge on the structure and biosynthesis of peptide cannabinoids. We also provide new insights on their activation and modulation of cannabinoid receptors.

## The Endocannabinoid System at a Glance

### Cannabinoid Receptors

The biological effects of cannabinoids are mainly mediated by two members of the G-protein-coupled receptor family (GPCRs), CB1 and CB2. However, new families of receptors that can bind and are modulated by cannabinoid compounds are being described (Di Marzo, 2018). These non-canonical cannabinoid receptors are a highly heterogeneous and complex group of seven-pass-transmembrane domain and nuclear receptors, as well as ion channels and transmitter-gated receptors. Respectively, orphan GPCRs like GPCR 55 (GPR55), GPR18 and GPR110 (Ryberg, et al., 2007; Lauckner et al., 2008; McHugh et al., 2009; Lee et al., 2016), nuclear receptor family of proliferator-activated receptors (PPARα PPARγ PPARδ), ionotropic receptors such as the transient receptor potential cation channel subfamily V and subfamily A members (TRPV1, TRPV2, TRPV3, TRPV4 and TRPA1) (De Petrocellis et al., 2017), transient receptor potential cation channel subfamily M member 8 (TRPM8) (De Petrocellis et al., 2017) and ion channels for neurotransmitters, such as serotonin 1A receptor (5-hydroxytryptamine 5HT1A) (Resstel et al., 2009), glycine and the GABAA receptor (GABA_A_R) (Hanuš et al., 2016).

In humans, CB1 was found to be a protein consisting of 472 amino acids and to be encoded by the gene CNR1 on chromosome 6 (Gerard et al., 1990). CB2 was first characterized in cells of the immune and hematopoietic system (Galiegue et al., 1995). This receptor is encoded by the gene CNR2 located on chromosome 1 (Yao and Mackie, 2009). It was cloned and characterized as a 360 amino acids transmembrane protein, sharing only 44% of the CB1 amino acid sequence (Munro et al., 1993). Along with the canonical long form of the CB1, two additional N-terminal shorter isoforms have been reported. While the full-length CB1 dominates in brain and skeletal muscle, the shorter isoforms show a higher expression level in liver and pancreatic islet cells, where it is involved in metabolism (González-Mariscal et al., 2016). In addition, two isoforms of CB2 (a and b) have been identified, with CB2a predominantly expressed in testis, but also in low levels in brain, whereas CB2b is mainly expressed in spleen (Liu et al., 2009).

**FIGURE 1:**
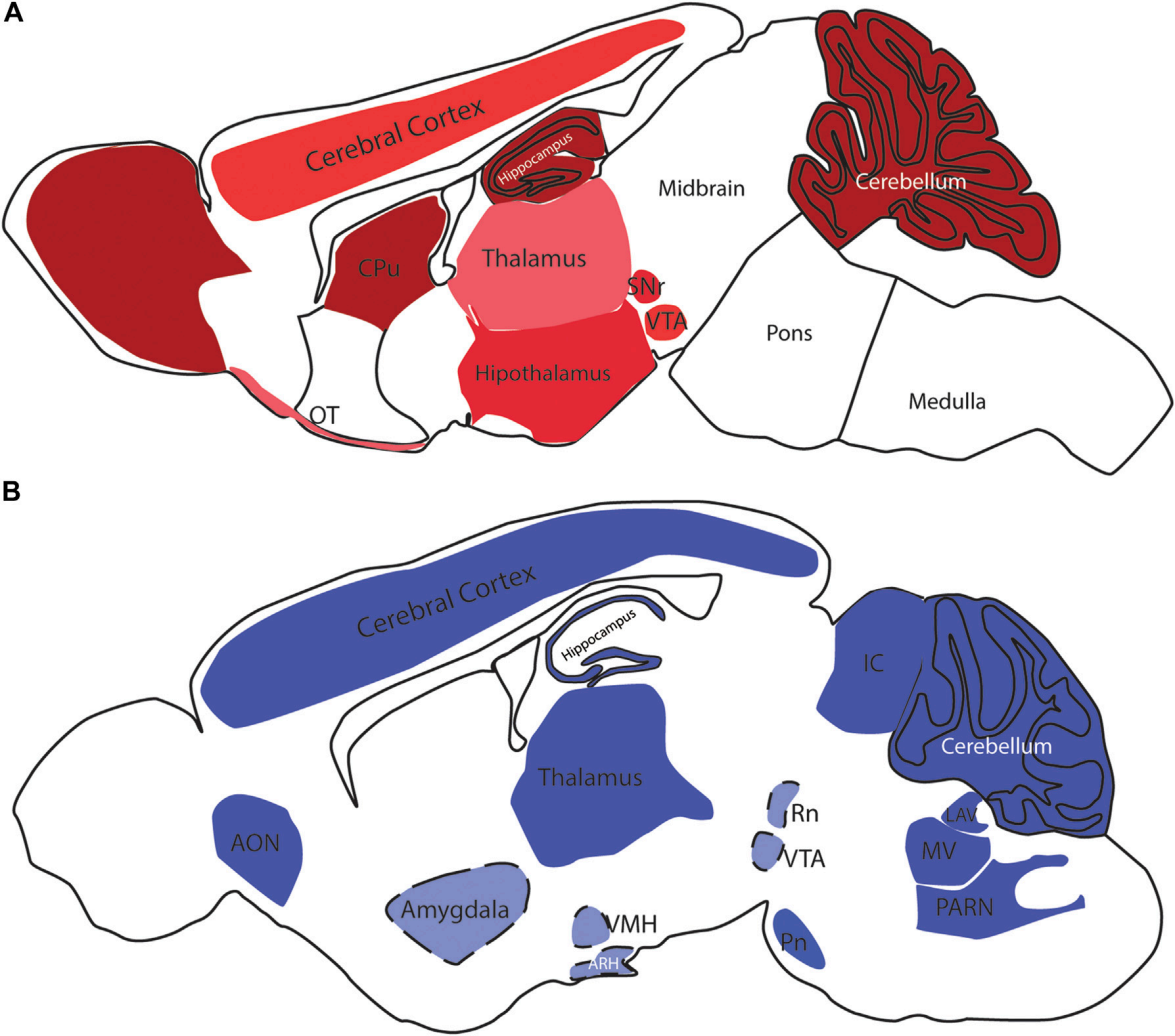
CB1 and CB2 receptors in the rodent brain. In **(A)**, schematic distribution of CB1 receptor sites in the mouse brain. The color code demonstrates the differential expression, ranging from the low (pink) to high levels (dark red); the brain depicted in **(B)** is from mouse, but some of the expression sites represented here were described in the rat brain. Light blue colored areas with dashed lines represent areas that were not visible in this sagittal section level. OB- Olfactory Bulb, CPu—Caudate Putamen, Acb—Nucleus Accumbens, SNr—Substantia Nigra pars compacta, VTA—Ventral Tegmental Area, PAG—Periaqueductal gray, OT—Olfactory Tubercle, NTS—Nucleus of the solitary tract, AON—Anterior olfactory nucleus, VMH—Ventromedial Hypothalamic nucleus, ARH—Arcuate Hypothalamic nucleus, IC—Inferior colliculus, RN—Red nucleus, PN—Pontine nucleus, LAV—Lateral vestibular nucleus, MV—Medial vestibular nucleus, PARN—Parvocellular reticular nucleus.

CB1 is the most abundant GPCR in the CNS and is widely distributed (Mackie, 2005) (Figure 1) mostly in neurons but also in nonneuronal cell types and stem cells/progenitors (Navarrete and Araque, 2008; Mato et al., 2009; Han et al., 2012). Contrary to the initial belief that CB2 was exclusively present in the periphery, particularly in the immune system, in the CNS, CB2 is highly expressed in microglia (Walter et al., 2003) as well as in astrocytes, oligodendrocytes, neural stem/progenitor cells, vascular elements in the brain (Maccarrone et al., 2014) and even neurons (Molina-Holgado et al., 2002; Stella, 2004; Sheng et al., 2005; Galve-Roperh et al., 2013). CB2 was first identified in peripheral nervous system (PNS) neurons (Griffin et al., 1997; Ross et al., 2001) and later in brainstem and cerebellar neurons (Skaper et al., 1996; Van Sickle et al., 2005). Expression of CB2 was observed in some other areas of the CNS (Gong et al., 2006; Onaivi et al., 2006; Jhaveri et al., 2008; Suárez et al., 2009; Lanciego et al., 2011; Xi et al., 2011) (Figure 1).

There is also evidence supporting the continuous internalization of plasma membrane-localized CB1 forming organelles like late endosomal/lysosomal compartments (Thibault et al., 2013). This organelle CB1 population had distinct pharmacological properties from their plasma membrane counterparts (Rozenfeld and Devi, 2008). Another subpopulation of subcellular CB1 is expressed in mitochondrial membranes and was found to modulate mitochondrial respiration (Bénard et al., 2012). Recently, it was also shown that mitochondrial CB1 regulates memory processes via modulation of mitochondrial energy metabolism linking mitochondrial activity to memory formation (Hebert-Chatelain et al., 2016).

In summary, cannabinoid receptors and non-canonical cannabinoid receptors are highly heterogeneous. Because eCBs interact with these multiple receptors, it is difficult to directly activate CB1 and CB2 receptors without affecting other receptors. It is also hard to activate a specific target, as well as a specific signaling pathway. Since there is heterogeneous expression of cannabinoid receptors in different cellular environments (eg cell types, tissues, organ), the same cannabinoid ligands may produce diverse biological responses through both CB and non-canonical CB receptors, triggering different signaling pathways. In addition, it is important to underscore that cannabinoid receptors expressed in different cell types have different effects (Busquets-Garcia et al., 2015), which may provide a cell specific approach to treatment. Thus, expanding research in cannabinoid signaling in its subcellular, cellular and regional levels may yield a plethora of possibilities unexplored in medicine. Therefore, to fully identify and understand the biological effects of cannabinoid function, it is also necessary to understand the cellular and anatomical contexts in which differential expression patterns of cannabinoid receptors and ligands may favor one or other signaling pathway.

### Lipid Endocannabinoids

Endogenous cannabinoids are small lipid-derived signaling molecules, not restricted to the CNS, since they have also been detected throughout the entire body (Zou and Kumar, 2018). Moreover, in the CNS, the production of eCBs is not restricted to neurons, since they are also produced in astrocytes, microglia and neural stem/progenitor cells (Walter and Stella, 2004; Maccarrone et al., 2014). These fatty acid derivatives cannot be packaged into vesicles, but are directly synthesized from membrane phospholipids. Thus, their production is mainly thought to be “on-demand” and “use-dependent” in a Ca^2+^-dependent manner in response to depolarization, or after activation of a G protein-coupled receptors.

**FIGURE 2:**
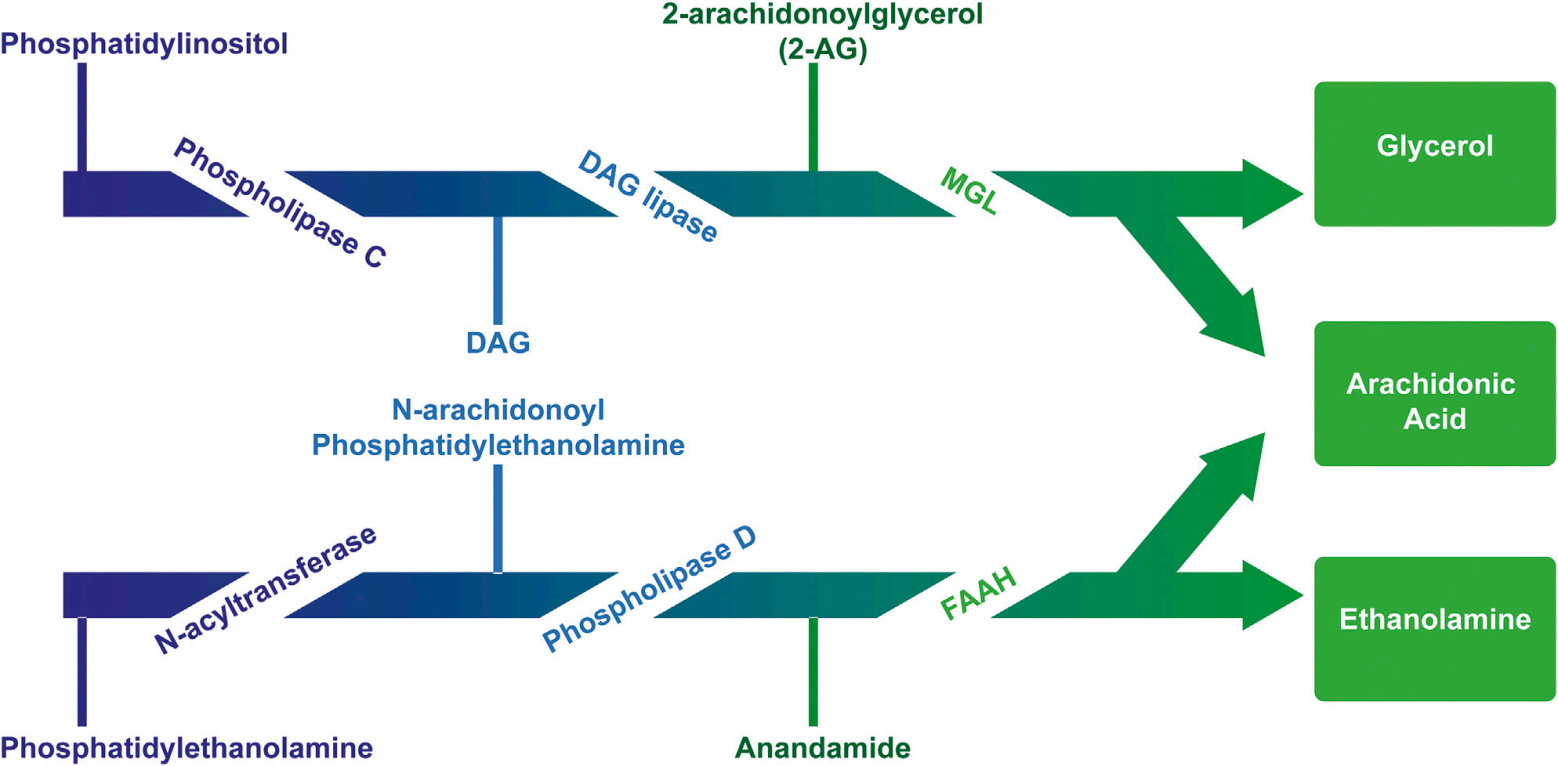
Major pathway for the synthesis and degradation of the endocannabinoids AEA and 2AG. AEA is mainly synthesized from membrane lipids “on demand.” AEA synthesis includes the transfer of arachidonic acid from 1-arachidonoyl-phosphatidylcholine (PC) to phosphatidylethanolamine (PE) catalyzed by N-acyltransferase (NAT), thus generating the AEA precursor, N-arachidonoylphosphatidylethanolamine (NAPE) subsequently cleaved by NAPE-specific phospholipase D (NAPE-PLD) releasing AEA and phosphatidic acid (PA). The precursor N-acyl-phosphatidylethanolamine can also be hydrolyzed by phospholipase A2 and the product, N-acyl-lysophosphatidylethanolamine, is then metabolized by liso-PLD, forming several N-acylethanolamines, and among them AEA. There are other relevant biosynthetic pathways for anandamide, such as the α/β-hydrolase domain 4, glycerophosphodiesterase 1, PLC and protein tyrosine phosphatase non-receptor type 22 (Maccarrone, 2017) (not shown). The 2-AG is synthesized from the diacylglycerol hydrolysis (DAG) by a selective lipase DAG for the sn-1 position. DAG can be directly converted into 2-AG through the action of two Ca^2+^-sensitive sn-2-selective DAG lipases, i.e. DAGL-α and DAGL-β. On the other hand, DAG can be generated from the hydrolysis of either phosphoinositides (PI), catalyzed by a PI selective phospholipase C (PI-PLC), or phosphatidic acid (PA), catalyzed by a phosphohydrolase PA. Two different pathways can degrade eCBs: hydrolysis and oxidation (Robbe et al., 2001). Enzymes which catalyze the first pathway include fatty acid amide hydrolase (FAAH) for anandamide and monoacylglycerol lipase (MGL) for 2-AG. The second route involves the enzymes cyclooxygenase (COX) and lipoxygenase (LOX), which induce the oxidation of the arachidonic fraction of endocannabinoids.

The first endogenous ligand for cannabinoid receptors was discovered and named “anandamide,” based on Ananda, the Sanskrit word for “joy, bliss, delight”: the arachidonoyl ethanolamide or anandamide (AEA) (Devane et al., 1992). Briefly after, the second one was identified: the 2-arachidonoyl glycerol (2-AG) (Mechoulam et al., 2009; Sugiura et al., 1995). They are originated from arachidonic acid-derived lipids linked to glycerol (diacylglycerol - DAG) or amines (N acyl amides) (Ahn et al., 2008; Pertwee, 2010). Despite both anandamide and 2-AG contain arachidonic acid, their routes of synthesis and degradation are almost completely distinct and are mediated by different enzymes (Pacher et al., 2006) (Figure 2). Buczynski and Parsons (2010) reported the whole brain concentration of 2-AG amounts to 12 nmol g^−1^, which was much higher than AEA, that had the concentration of 19 pmol g^−1^.

It has been observed that the production and synthesis of AEA and 2-AG can occur independently within the cell. Neurons produce different eCBs in a receptor-dependent manner. The activation of NMDA receptors in cortical neurons increases 2-AG levels without affecting the formation of AEA, which requires the simultaneous activation of NMDA receptors and α-7 nicotinic receptors (Stella and Piomelli, 2001). Also, the electrical stimulation of hippocampal slices increases the release of 2-AG, but not AEA (Stella et al., 1997). In contrast, activation of dopamine D2 receptors increases levels of AEA and not those of 2-AG (Pan et al., 2008). Additionally, functional cross-talk between 2-AG and AEA signaling was reported, and recent findings suggest that 2-AG and AEA can be recruited differentially from the same postsynaptic neuron depending on the type of presynaptic activity (Puente et al., 2011; Lerner and Kreitzer, 2012). The eCB degradation pathways are segregated across the synapse, with the AEA-catabolic enzyme fatty acid amide hydrolase (FAAH) localized mainly in postsynaptic structures and the 2-AG-degrading enzyme monoacylglycerol lipase (MGL) located mainly on the presynaptic side (Gulyas et al., 2004; Blankman et al., 2007). The differences in efficacy and the spatial segregation of the degradation pathways for the two eCBs support the hypothesis that AEA and 2-AG have differential functional roles in eCB-mediated signaling in different brain regions or at different synapses within these brain regions.

The endocannabinoid actions are complex due to their lipophilic nature and the important feature that their precursors are present in lipid membranes and synthesized upon demand. Moreover, they are promiscuous mediators because they activate a large number of receptors and may overlap with other signaling pathways and metabolic processes. AEA behaves as a partial agonist for both CB1 and CB2 receptors (Reggio, 2010), and also binds transient receptor potential cation channel subfamily V member 1 (TRPV1) (Zygmunt et al., 2013) and GPR55 (Ryberg et al., 2007; Yang et al., 2016). Because 2-AG can activate CB1 and CB2 with their maximum response, it was characterized as a full agonist (Rockwell et al., 2006). In addition to cannabinol. id receptors, 2-AG modulates the nuclear fatty acid receptors—the peroxisome proliferator activated receptor-α and -γ (PPARα and PPARγ) (Rockwell et al., 2006; Raman et al., 2011), the GABA_A_ receptor (Sigel et al., 2011), adenosine A3 receptor (Lane et al., 2010), TRPV1 (Zygmunt et al., 2013), and GPR55 (Ryberg et al., 2007).

Other lipid groups are considered to be eCBs, such as the fatty acid ethanolamines, which are chemically similar to anandamide and biosynthesized and/or degraded by the same enzymes, i.e. N-oleoylethanolamine (OEA) and N-palmitoylethanolamine (PEA), the fatty acid primary amides, and the monoacylglycerol related molecules (Silvestri and Di Marzo, 2013) (Table 1). These lipid mediators often share receptors and/or catabolic enzymes with the classical endocannabinoids. The biosynthetic pathways of AEA and 2-AG can also interfere with the biosynthesis of other N-acylethanolamines and monoacylglycerols. These molecules can activate the receptor family PPAR, the G-protein-coupled receptor GPR119, the vanilloid receptor, and several ion channels, which may also be activated by “true endocannabinoids”. These lipids have been correlated with the regulation of fundamental processes including pain perception (analgesia), inflammation, sleep, and feeding behavior (Ezzili et al., 2010; Hansen, 2013).

**TABLE 1:** Lipid endocannabinoids, the chemical structure and their receptors.

Endocannabinoid	Chemical structure	Receptor activity
N-arachidonylethanolamide (anandamide)	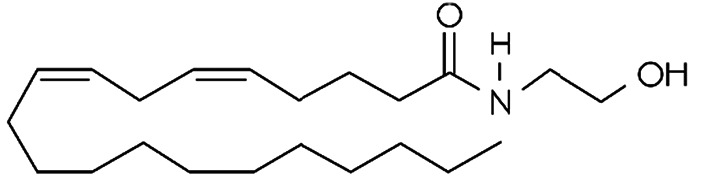	CB_1_ and CB_2_ partial agonist and also can bind TRPV_1_ and GPR55
2-Arachidonoylglycerol (2-AG)	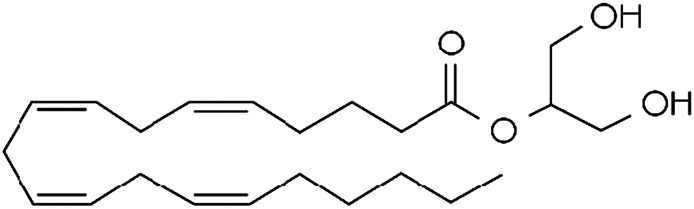	CB_1_ and CB_2_ complete agonist and also binds to PPARα and PPARγ
O-arachidonoyl ethanolamine (virodhamine)	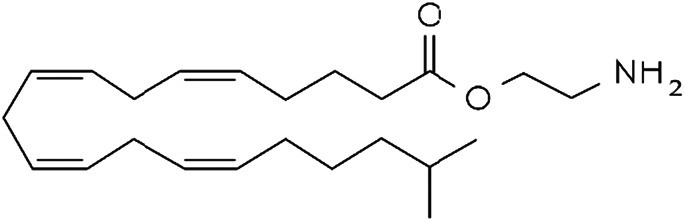	CB_1_ partial antagonist and CB_2_ agonist
2-Arachidonoylglycerol ether (noladin ether)	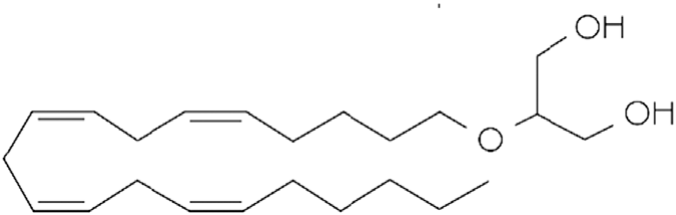	CB_1_, CB_2_ and GPR55 agonist and Trpv1 partial agonist
Dihomo-γ-linolenoyl ethanolamide)	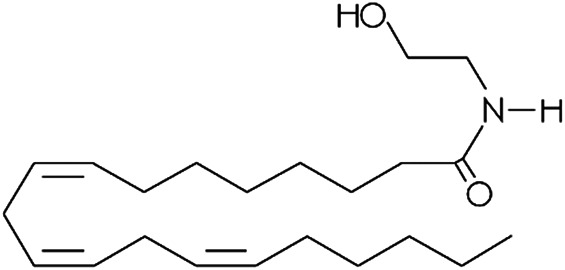	CB_1_ and CB_2_ agonist
N-arachidonoyl-dopamine (NADA)	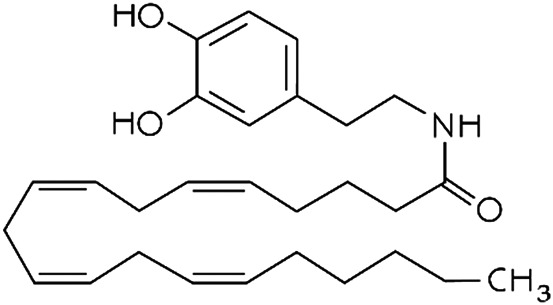	CB_1_ agonist and TRPV_1_ ligand
Cis-9,10-octadecenomide (oleamide)	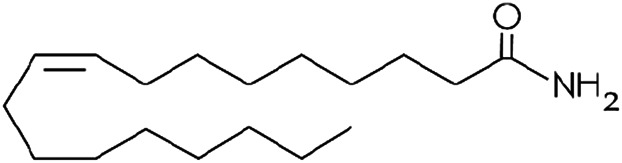	CB_1_ parcial agonist and CB_2_ agonist
N-palmitoyethanolamide (PEA)	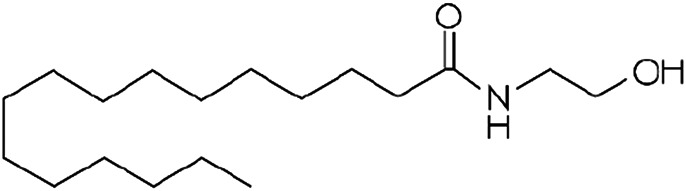	Binds to PPAR and GPR55
N-oleoylethanolamide (OEA)	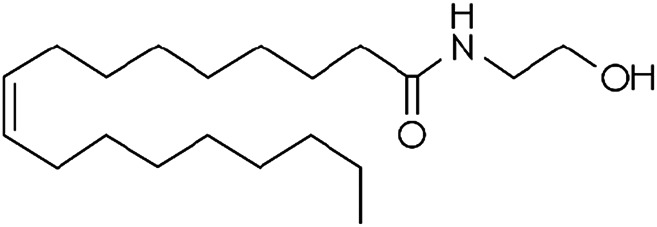	TRPV_1_ and PPAR agonist
Docosatetraenoylethanolamide (DEA)	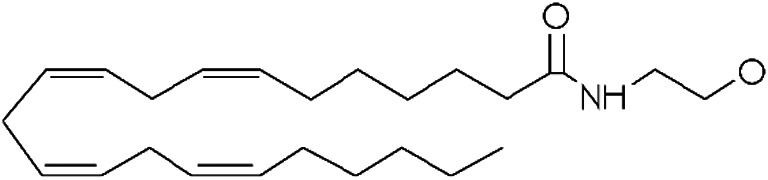	CB_1_ receptor

Regulation of neurotransmitter release constitutes a major physiological role of the ECS. eCBs are produced and released from postsynaptic neurons either by phasic (in an activity-dependent manner), or tonic (basal) conditions (Mackie, 2006; Di Marzo, 2008; Castillo et al., 2012). Both AEA and 2-AG are synthesized on the postsynaptic side of the synapse and then act retrogradely by activating presynaptic receptors. However, AEA may be synthesized presynaptically, therefore acting at postsynaptic TRPV1 (Chávez et al., 2010; Grueter et al., 2010). In addition, it has been observed that AEA binds to TRPV1 on the same intracellular binding site as capsaicin and the degradation by fatty acid amide hydrolase limits anandamide activity on these receptors (De Petrocellis et al., 2001). AEA acting on TRPV1 mediates a postsynaptic form of LTD. through increase in AMPA receptor reuptake in the medium spiny neurons of the nucleus accumbens (Grueter et al., 2010), and in dentate granule cells (Chávez et al., 2010). TRPV1 receptors often have roles opposite to those of classical cannabinoid receptors. TRPV1 was thought to increase excitability of CNS neurons, as suggested by studies in epilepsy models (Bhaskaran and Smith, 2010; Sun et al., 2012). Recently, it was reported that presynaptic TRPV1 activation facilitates glutamate release. It was observed that metabotropic NMDAR-Panx1 suppressed facilitated glutamate release during low-frequency synaptic stimulation because postsynaptic Panx1 maintained low levels of AEA, thus limiting activation of presynaptic TRPV1 channels (Bialecki et al., 2020).

In addition to retrograde and anterograde actions in synapses, AEA and 2-AG signal both in an autocrine and paracrine manner. Endocannabinoids produced by neurons bind to the cannabinoid receptors expressed in astrocytes and microglia, modulating a neuron-glia signaling pathway (for reviews see Katona and Freud, 2008; Castillo et al., 2012; Pertwee 2010). The stimulation of astrocytic CB1 receptors causes glial release of glutamate and neuronal metabotropic glutamate receptor 1 (mGluR1) activation in hippocampus, producing synaptic potentiation that may affect neuronal/synaptic function (Navarrete and Araque, 2008; Navarrete and Araque, 2010; Han et al., 2012). Navarrete and Araque (2008) showed that eCBs increases astrocyte Ca^2+^ levels evoked by CB1 activation in a G_q/11_ dependent-manner, a finding that was also demonstrated in human brains (Navarrete et al., 2013). The activation of this signaling pathway lead to glutamate release from astrocytes, which evoked slow inward currents on adjacent CA1 pyramidal neurons of hippocampus mediated by NMDA receptors activation, but also increased the potentiation of synaptic transmission in distant heteroneuronal synapses via pre-synaptic type 1 metabotropic glutamate receptor activation (Navarrete and Araque, 2010). Recently, Hegyi et al. (2018) also demonstrated that this evoked Ca^2+^ transients mediated by CB1 induced the release of 2-AG in rodent spinal cord astrocytes, a novel endocannabinoid induced endocannabinoid release mechanism. On the other hand, the release of astrocyte glutamate in the sensory cortex has been shown to activate pre-synaptic NMDAR leading to long term depression (LTD.), which may be important for synaptic plasticity changes on sensory information processing (Griffin and Nevian, 2012). Therefore, the ECS has a significant role on neuron-astrocyte communication by inducing the release of gliotransmitters which, in turn, act on a dual manner, potentiating or depressing synapses to promote a variety of synaptic changes that may be critical for neural plasticity.

Another crucial aspect of the ECS function is its participation in controlling neuroinflammation and neurodegenerative processes. The declines in neural function are likely due to the concerted involvement of different insults including protein misfolding, neuroinflammation, excitotoxicity, oxidative stress and mitochondrial dysfunction and all these pathological processes appear to be modulated by the eCB signaling system (Cassano et al., 2017). Particularly, the modulation of astrocyte and microglia functions by the endocannabinoid system emerges as an interesting pharmacological tool, especially in neuroinflammatory processes. Astrocytes and microglial cells are the primary effectors of reactive gliosis, changing their phenotype and functions in response to several CNS insults (Kozela et al., 2017). Several models of neuroinflammation have been reported to be beneficially modulated by cannabinoids. Wang et al. (2018) demonstrated that 2-AG not only suppressed glutamine synthetase (GS) expression, an astrocyte enzyme involved in inflammation, in the early phase of lipopolysaccharide (LPS) exposure, but also reversed its well-known decreased expression in the late phase of the exposure, contributing to astrocyte protection and to a reduced neuroinflammatory condition induced by LPS. Also, 2-AG has been shown to cause a decrease in acute expression of IL-1, IL-6 and TNF-a pro-inflammatory cytokines and an increase in neuroprotection via CB1 activation on a model of head injury (Panikashvili et al., 2006). Finally, cannabidiol (CBD), derived from *Cannabis sativa*, was also shown to modulate neuroinflammatory conditions. Atalay et al. (2020) reported CBD antioxidant effects by reacting oxygen species (ROS) generation inhibition, inducing antioxidative enzyme expression and indirect effects by increasing anandamide levels leading to reduced oxidative stress and decreased inflammation. Thus, it is clear that cannabinoids have a major role in the regulation of CNS inflammatory conditions and oxidative processes mainly by modulating astrocyte and microglial functions.

Since eCBs have been reported to promote neuroprotection against demyelination, several studies attempted to understand the role of the ECS in myelinating disorders. Tomas-Roig et al. (2020) demonstrated that a WIN55,212–2 dose of 0.5 mg/kg induced myelin repair following a demyelinating insult by cuprizone injection. However, an increased dose of 1.0 mg/kg caused the opposite, severe demyelination accompanied by limited potential of myelin repair. Also, Bradshaw and collaborators (2019) have shown that 2-AG activated microglial cells to promote myelin debris phagocytosis in the *corpus callosum* in order to regenerate myelin sheath surrounding *callosum* fibers, a spontaneous turnover mechanism essential for maintenance of synaptic function. There is also novel evidence revealing improvement of demyelination in experimental autoimmune encephalomyelitis (EAE) model of multiple sclerosis via anti-inflammatory effects mediated by CB1 (Arévalo-Martín et al., 2003; Solbrig et al., 2010; Rossi et al., 2011; De Lago et al., 2012). Cannabinoids also regulate oligodendrocyte cell survival, proliferation and differentiation (Ilyasov et al., 2018). These findings highlight the relevance of the endocannabinoid system regulating oligodendrocyte renewal and survival, as well as myelination regulation and potential therapeutic approaches for demyelinating diseases.

To date, all of the endogenous ligands found to act at cannabinoid receptors are lipid-derived fatty acid congeners, although they can be structurally diverse within that group. Recently, it was found that cannabinoid receptor activity can be modulated by peptides derived from the hemoglobin protein. There is increasing evidence that hemoglobin related peptides act as modulators of the cannabinoid system providing new pharmacological and biological properties on cannabinoid signaling. We will now briefly describe the current knowledge on the peptide cannabinoids, a new player on the already complex ECS.

### Cannabinoid Receptors Signaling Pathways

Endocannabinoids released postsynaptically retrogradely activate cannabinoid receptors in the presynaptic terminal, modulating synaptic transmission by suppression of transmitter release in a transient or long-lasting manner at both excitatory (glutamate) and inhibitory (GABA) synapses throughout the CNS, thereby mediating various forms of long- and short-term plasticity (Freund et al., 2003; Chevaleyre et al., 2006; Castillo et al., 2012; Katona and Freund, 2012). For instance, CB1 receptors regulate neurotransmission in different ways. For short-term plasticity, the mechanism involves direct G protein-dependent (via βγ subunits) inhibition of presynaptic Ca^2+^ influx through voltage-gated Ca^2+^ channels (VGCCs) (Brown et al., 2003). For long-term plasticity, CB1 modulates the levels of intracellular cAMP and therefore regulates the phosphorylation of protein kinase A to produce changes in cellular activity (Childers and Deadwyler, 1996). The inhibition of the cAMP/PKA pathway, besides regulating neurotransmitter release, also modulates synaptic plasticity and neuronal remodeling (Basavarajappa, 2007).

More recently, it has been shown that cannabinoids modulate the release of other neurotransmitters besides glutamate and GABA such as dopamine (Lau and Schloss, 2008; Bloomfield et al., 2016), noradrenaline (Busquets-Garcia et al., 2016), acetylcholine (Goonawardena et al., 2010), and serotonin (Haj-Dahmane and Shen, 2011). ECS have been related to several CNS regions and brain functions: memory and learning, decision making and emotional behavior, regulation of voluntary movements and movement learning, motor control and spatial coordination, reward, anxiety and stress, fear, pain sensation, sleep control of light/dark cycles, eating behavior (Mechoulam and Parker, 2013), and addiction (Maldonado et al., 2006).

CB1 and CB2 receptor signaling is pleiotropic and depends on the cellular type, anatomical localization and cellular functional state. These receptors primarily couple to G_i/o_ protein inhibiting adenylyl cyclase and voltage-gated Ca^2+^ channels while they activate inwardly rectifying K^+^ channels, phospholipase C, phosphatidylinositide-3-kinase (PI3K) and mitogen-activated protein kinases (MAPK) (Howlett and Shim, 2013; Ibsen et al., 2017). Besides the important role in regulating neurotransmitter release, cannabinoids are also involved in the control of cell cycle, cell metabolism, cell survival, cell fate and apoptosis. They activate different MAPK and PI3K cascades (eg the PI3K/Akt and the extracellular signal regulated kinase [ERK]). CB1 and CB2 activation of the PI3K/Akt pathway regulates nuclear transcription factors such as MAPK families, ERK1 and ERK2, c-Jun N-terminal kinase (JNK) and the p38 mitogen-activated protein kinase. They also modulate the glycogen synthase kinase 3 (GSK3), mammalian target of rapamycin complex 1 (mTORC1), the generation of sphingolipid-derived signaling mediators and cell death pathways (eg caspases activation and the endoplasmic reticulum stress response) (Kofalvi, 2008; Howlett and Abood, 2017).

On the other side, CB1 is promiscuous in relation to its G protein coupling. Besides coupling to G_i/o_, CB1 can also couple with G*α*_s_- and G*α*_q_-dependent signaling under some conditions. Furthermore, it was observed that different agonists activate different G subunit such as G*α*_z_, G*α*_q/11_, and G*α*_12/13_ (Diez-Alarcia et al., 2016). The consequences of activating G*α*_s_, and G*α*_q_ heterotrimers have been described for many GPCRs, and this signaling seems similar for CB1 and CB2. Contrary to G_i/o_, G*α*_s_ stimulates adenylyl cyclase and activates Ca^2+^ channels, while G*α*_q_ couples to phospholipase C and promotes the release of intracellular calcium (Ibsen et al., 2017). Thus, cannabinoid receptors are capable to couple to different families of G proteins, suggesting that different intracellular responses may be activated depending on the ligand, in a mechanism known as functional selectivity or biased agonism (Bosier and Hermans, 2007). Biased agonism implies that structurally different ligands will induce diverse conformations of the receptor, which may then favor one of the possible signaling pathways over the others. Recently, new ways of cannabinoid receptor modulation have been proposed, such as internalization, desensitization, heteromerization and allosteric modulation, evidencing the complexity of the ECS on its signaling pathways.

### Modulation of the CB1/CB2 Signaling Pathways

Besides recruiting different G proteins, cannabinoid receptors can recruit other proteins for signaling, most prominently β-arrestin-1 and -2, which mediate receptor desensitization, regulation of receptor sensitivity to acute agonists and receptor internalization (Breivogel et al., 2008; Nguyen et al., 2012; Raehal and Bohn, 2014). Also, β-arrestin-1 and -2 support the continuous internalization of plasma membrane-localized CB1 by forming organelles such as endosomal and lysosomal compartments (Thibault et al., 2013). The organelle CB1 population has distinct pharmacological properties from their plasma membrane counterparts (Rozenfeld and Devi, 2008).

In addition, it has been discovered that the CB1 receptor can form homo and heterodimers with other GPCRs, such as dopaminergic receptors (Kearn et al., 2005), adenosine receptors (Carriba et al., 2007) opioid receptors (Hojo et al., 2008) and orexin receptors (Ward et al., 2011). Also, Kargl et al. (2012) have reported that GPR55 co-immunoprecipitated with CB1. It was also recently shown that CB1 can form homodimers and heterodimers with CB2 in a variety of brain regions (Callén et al., 2012).

Another form of modulating receptor signaling is via allosteric modulation. Endogenous molecules of diverse chemical nature have been identified as CB1 allosteric modulators. One of these molecules is lipoxin A4, an oxygenated derivative of arachidonic acid involved in immune system regulation known as a potent endogenous anti-inflammatory mediator. Lipoxin A4 was shown to be a CB1 positive allosteric modulator both *in vitro* and *in vivo* (Pamplona et al., 2012). This lipid enhances CB1 receptor binding of AEA [^3^H]CP55,940 and WIN55,212–2, thereby potentiating their signaling and behavioral effects. It also selectively potentiated AEA response vs. 2-AG in HEK293-CB1 cells. Surprisingly, Straiker et al. (2015) reported that lipoxin A4 attenuated CB1 response of 2-AG-mediated depolarization-induced suppression of excitation (DSE) suggesting a negative modulation (Straiker et al., 2015). Another endogenous molecule is pregnenolone, a steroid and precursor/metabolic intermediate that was identified in the biosynthesis of most of the steroid hormones. It was reported that tetrahydrocannabinol (THC) increases pregnenolone levels in different brain regions. In turn, this steroid decreases THC-induced signaling showing a CB1 negative allosteric modulation. Moreover, it was shown to decrease certain effects of cannabinoids such as food intake and memory impairment (Vallée et al., 2014; Khajehali et al., 2015). Nevertheless, Straiker and collaborators showed that pregnenolone failed to modulate 2-AG synaptic transmission (Straiker et al., 2015). Besides these endogenous molecules, plant-derived cannabinoids can also act as allosteric modulators. Recent studies suggest that cannabidiol (CBD) can act as CB1 negative allosteric modulator and regulates THC- and 2-AG-dependent CB1 internalization, β-arrestin recruitment, phospholipase C activation and ERK1/2-phosphorylation (Laprairie et al., 2015).

Cannabinoid receptor-mediated signaling not only depends on direct receptor activation effectors, but also on larger signaling complexes, which include other modulatory proteins-like mediators of desensitization and internalization. Moreover, CB receptors form both homo- and heterodimers and undergo allosteric modulation, which correlates to receptor functionality (Ferré, 2007; Franco et al., 2008). Also, this modulation may modify the signaling properties of a given ligand, affecting the selectivity of the interaction between receptor and G protein, resulting in potentiation, attenuation or even coupling with another G protein. These modulatory properties of cannabinoid receptors have a number of potential advantages, including allowing improved spatiotemporal regulation of an endogenous ligand, besides providing a mechanism for a more precise control of downstream pathways.

## Peptide Endocannabinoids

### Structure, Self-Aggregation and Receptor Binding

The hemopressin peptides are a family of polypeptides derived from either α or β hemoglobin chains with a molecular weight range of 1.4–2.6 kDa (Gomes et al., 2009; Bauer et al., 2012). They result from the cleavage of the α-chain hemoglobins in the amino acid position 96 to 119. The shorter one is hemopressin (Hp), a 9-residue amino acid peptide (PVNFKLLSH) derived from hemoglobin cleavage at amino acid positions 96–104. Hp is a peptide with the molecular formula C53H77N13O12 and molecular weight of 1,088.3 g/mol. The amino acid sequence of Hp is well conserved across mammalian species (Heimann et al., 2007). However, the sequence alignments of Hp from various species differ only at amino acid position 100 of the α 1-globin chain, where F in rat is replaced by L in human, pig, and cow sequences. Hp acts as substrate for three proteases (endopeptidase ep24.15, endopeptidase 24.16 and angiotensin-converting enzyme ACE), where it can be cleaved by the ep24.15 and ep24.16 endopeptidases generating shorter fragments (PVNF, PVNFK, PVNFKF and PVNFKFL) (Dale et al., 2004; Dale et al., 2005). Initially, only the peptides with six and seven amino acids were thought to have biological activity (Dale et al., 2004; Dale et al., 2005; Bomar and Galande, 2013). However, it was recently shown that oral administration of the hemopressin fragment NFKF prevented or altered seizures in a pilocarpine-induced epileptic seizure model (De Araujo et al., 2019).

Besides Hp, other N-terminal extended hemopressin peptides have been isolated and have different lengths, ranging from 11 to 23 amino acids (Gomes et al., 2009; Bauer et al., 2012). However, Hp is not an endogenous peptide, but rather an extraction artifact, a cleavage product of longer endogenous peptides. Acidic conditions were used in the original extraction of Hp, since the N-terminal extended residues of hemopressin peptides are D-P, which are some of the most labile bonds especially under acidic conditions (Marcus, 1985). It was argued that this acid extraction facilitates the cleavage of this bond on RVD-PVNFKLLSH (RVD-Hp) and VD-PVNFKLLSH (VD-Hp) to form Hp (Gomes et al., 2009; Gelman et al., 2010; Gelman and Fricker, 2010; Bauer et al., 2012). N-terminally extended hemopressin peptides RVD-Hp, VD-Hp and VD-Hpβ (VDPENFRLLCNM) (derived from the β-chain hemoglobin) were identified using an acid-free method. RVD-Hp and VD-Hp are twelve and eleven residue amino acid peptides derived from the cleavage of the α-chain hemoglobin at the amino acid positions 93–104 and 94–104, respectively, and VD-Hpβ is an eleven-residue amino acid peptide derived from the cleavage of the β-chain hemoglobin at the positions 99–110.

The physiological activity-structure relationship of these hemopressin peptides is still little known. Pioneering data using circular dichroism and nuclear magnetic resonance spectroscopy (NMR) showed that hemopressin is characterized by regular β-turn structures in the N3-H9 segment, whereas PVNFKF (Hp 1–6) showed a regular β-turn conformation in the N3-F6 segment, revealing that this shorter fragment adopted a different conformation than the nonapeptide. However, in both peptides, regular turn structures are present in the N3-F6 segment (Scrima et al., 2010). In addition, Bomar et al. (2012) also reported that Hp adopts these extended β-like structures in the presence of 25% trifluoroethanol (TFE) (Bomar et al., 2012). However, a recent work did not observe the same finding, but rather a helical region in Hp and RVD-Hp structure. Recently, it was shown that Hp and RVD-Hp are unstructured in aqueous environment and the presence of low polarity environment increased the content of α-helical structures. At acidic pH both peptides resembled the native conformation at the N-terminal region of the helix G of hemoglobin and presented a helical region at the C-terminus. Hp showed a helical region between residues 6 and 11, whereas RVD-Hp exhibited a helix spanning from V5 to S11. At neutral pH, the helical region was longer, but slightly shifted toward the N-terminus in RVD-Hp, whereas it was reduced to a single turn in Hp. Hp showed a helical region in the stretch N6-L10 and RVD-Hp presented a helical region spanning from D3 to S11. Hence, it was proposed that these helical conformations may resemble the peptide bioactive structure *in vivo* (Emendato et al., 2018).

Furthermore, it has also been observed that depending on the vehicle characteristics at high concentrations Hp can self-assemble and form fibril structures (Bomar et al., 2012). In addition, it was observed that larger peptides such as RVD-hp are more stable to degradation than smaller peptides and also less susceptible to self -assemble (Bomar and Galande, 2013). It was observed that Hp form dimers or oligomers and these aggregates and be retained on 2 kDa dialysis cassettes following 24 h of dialysis against phosphate-buffered saline (PBS) (Gomes et al., 2010). In addition, hemopressin was shown to self-assembly into nanostructure fibrils at physiological pH (1 mM peptide in 25 mM phosphate, 50 mM NaCl, pH 7.4). It was observed that Hp forms amyloid-like nanostructured fibrils, which may precipitate in the solution (Bomar et al., 2012). However, this fibrillation was not detected for RVD-Hp in similar conditions. It was proposed that the chirality of V2, the side chain of N3, L7, and the C-terminal carboxylic acid are among the major contributors toward Hp aggregation (Song et al., 2015). Using an ultrafiltration approach combined with a subsequent quantification of hemopressin peptides by C-ELISA with a specific monoclonal antibody against the C-terminal part of the peptides, it was observed that RVD-Hp and the 23-residue amino acid peptide formed an aggregate in a concentration-dependent manner. At high concentrations (1 µM), the peptides formed lower molecular weight aggregates such as dimers or trimers with approximately 2 KDa weight (Bomar et al., 2012). However, Emendato et al. (2018) did not find aggregation nor precipitation of Hp and RVD-Hp at different polarity and pH conditions at high concentration (Emendato et al., 2018).

The first six amino acids of the nonapeptide (PVNFKF) were found to be required for CB1 binding and the deletion of C-terminal three residues did not affect receptor recognition (Heimann et al., 2007). In addition, it was proposed that regular turn structures in the central portion of Hp and PVNFKF are critical for an effective interaction with the receptor. Recently, using a molecular docking analysis, it was observed that the smallest peptide NFKF, which have four-central amino acids of Hp, had a better Goldscore to bind CB1 than the antagonist AM6538, cannabidiol, and rimonabant (De Araujo et al., 2019). It was proposed that Hp cannot penetrate into the ligand binding pocket of CB1 and it can only bind to the external binding site of CB1 (Scrima et al., 2010). However, recently, docking studies have shown that the NMR conformation of Hp matched that of taranabant (inverse CB1 agonist) binding pocket, suggesting a similar bioactivity of Hp and rimonabant (Heimann et al., 2007; Emendato et al., 2018). The N-terminal extended hemopressin peptides are characterized as allosteric modulators and their nuclear magnetic resonance conformation showed that the structure of RVD-Hp is compatible with the binding site at one of the previously proposed allosteric sites on CB1 (Emendato et al., 2018).

Since hemopressin peptides do not cross the plasma membrane because of their peptide nature, they cannot activate these intracellular receptor subpopulations. This was confirmed both by using FACS analysis and measurements of intracellular fluorescence of cells treated with a fluorescent derivative of hemopressin in permeabilized cells and non-permeabilized cells, which revealed a lack of intracellular fluorescence in the latter. Furthermore, the spectrometric analysis of extracts of hippocampal sections incubated with Hp and the lipophilic CB1 antagonist AM251 did not show the intracellular presence of hemopressin (Bénard et al., 2012). To investigate the functionality of subcellular CB1 receptor, Rozenfeld and Devi (2008) stimulated neuro2A cells with the lipophilic CB1 agonist WIN55,212-2, which mediates ERK phosphorylation. They observed that the preincubation with the lipophilic CB1 receptor antagonist rimonabant (able to cross the membranes) completely inhibited WIN55,212-2-mediated ERK phosphorylation, while the pre incubation with Hp only partially inhibited it, indicating that Hp only can block the cell membrane CB1 receptor population (Rozenfeld and Devi, 2008). In addition, it was observed that the lipophilic CB1 agonist HU210 entered cells and modulated cellular respiration by activating mitochondrial CB1, while hemopressin was not able to inhibit the effect of HU210 confirming that Hp do not penetrate cells and thereby cannot activate mitochondrial CB1 (Bénard et al., 2012).

Therefore, even though a large body of data exists on the structure of these peptides *in vitro*, their behavior *in vivo* is still largely unknown. However, an important aspect about hemoglobin derived cannabinoids is that fragments of distinct size may influence receptors quite differently, firstly in terms of their CB1 receptor binding sites (orthosteric or allosteric) and, secondly, as a consequence on their receptor signaling capacities. Furthermore, evidence of their action in modulating other cannabinoid receptors was found and will be discussed later.

### Hemopressin and Related Peptides

**FIGURE 3:**
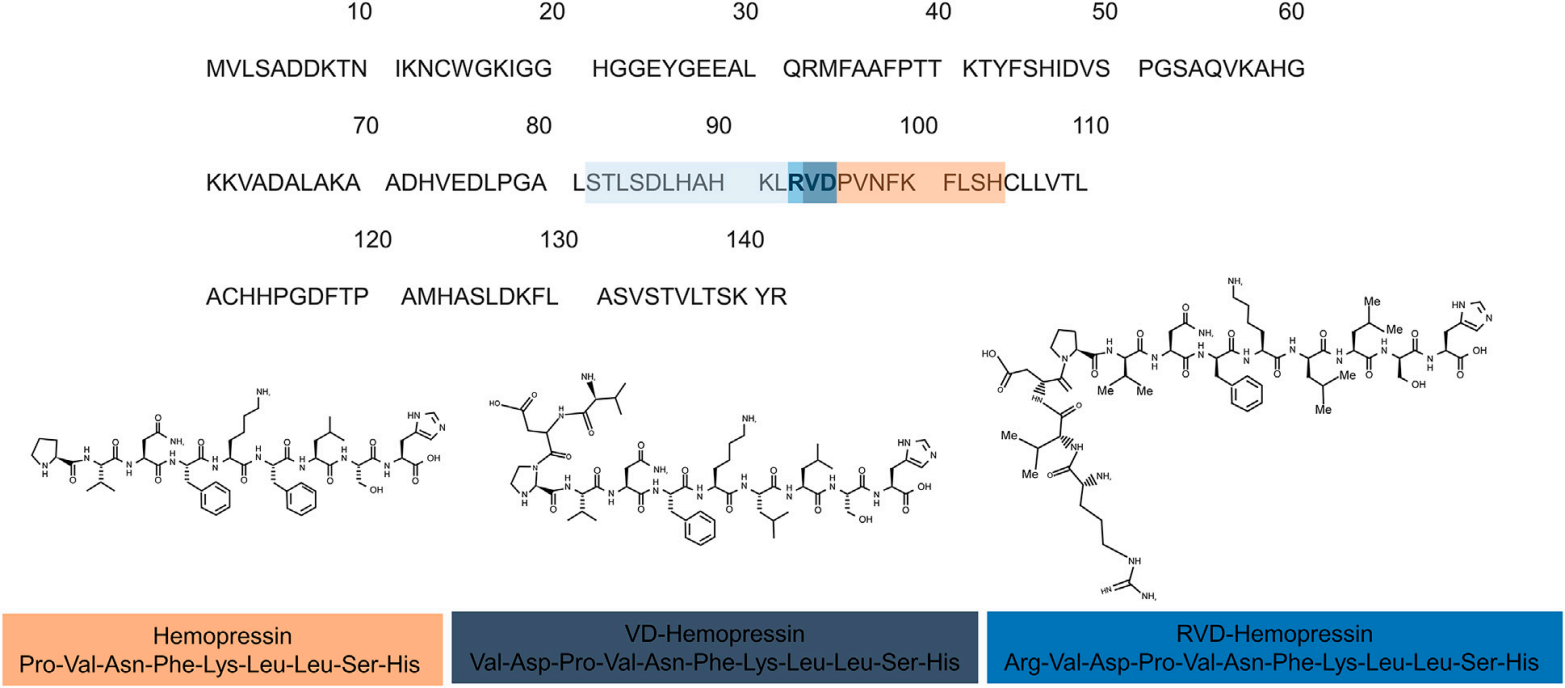
Hemopressin peptides are derived from the cleavage of hemoglobin α-chain. **(A)** Sequence alignments of the rat α-chain of hemoglobin. The sequence of hemopressin is underlined in the orange box the 2 (VD) and 3 (RVD) additional amino acid of VD-Hemopressin and RVD-Hemopressin respectively are in purple and dark blue. Light blue represents the additional amino acid residues of hemopressin peptide family. The number indicates the position of the amino acids. **(B)** Chemical structure of the hemopressin peptide (Hemopressin VD-hemopressin and RVD-Hemopressin). The hemopressin has 9-residue amino acid peptide with the amino acid sequence: Pro-Val-Asn-Phe-Lys-Leu-Leu Ser- His-OH while the VD-hemopressin has 11-residues (Val-Asp-Pro-Val-Asn-Phe-Lys-Leu-Leu-Ser-His-OH) and RVD-Hemopressin is an N-terminal extended hemopressin peptides with 12 amino acids sequence: Arg-Val-Asp-Pro-Val-Asn-Phe-Lys-Leu-Leu-Ser-His-OH.

The first description of hemopressin peptides was made in 2003 by Rioli and collaborators. When searching for novel bioactive peptides with an enzyme-substrate capture approach, commonly involved in neuropeptide metabolism in vertebrates, they reported the discovery of Hp (Rioli et al., 2003). Because of its hypotensive effect, it was named hemopressin (Dale et al., 2004). It has been shown that it has systemic vasodepressor activity in rat, decreases arterial pressure on both rabbit and mouse, dilates the rat systemic vascular bed and inhibits peripheral hyperalgesia (Dale et al., 2004; Blais et al., 2005; Lippton et al., 2006). Later, it was discovered that Hp is a selective ligand for CB1 receptor, explaining the mode of action of its previously described functions. Moreover, it was found that this peptide acts as a CB1 inverse agonist (Heimann et al., 2007). Heimann et al. (2007) also showed that the deletion of five amino acids from the C-terminal region of Hp affected CB1 receptor recognition, thereby influencing the Hp hypotensive and antinociceptive functions. Shortly after, Gomes et al. (2009), while searching for endogenous brain peptides, conducted the first report of the presence of N-terminal extended hemopressin endogenous peptides in brain, RVD-Hp and VD-Hp (Figure 3) (Gomes et al., 2009).

Bauer and collaborators (2012) used monoclonal antibodies against the C-terminal part of RVD-Hp to describe a whole family of N-terminal extended hemopressin peptides, which they refer as Pepcan-12 to -23, according to the peptide length (Figure 3). They reported that RVD-Hp (pepcan 12) is the shortest and most abundant of these peptides in the brain. Interestingly, they did not find Hp and VD-Hp in this approach (Bauer et al., 2012). In addition, they found that the average concentration of RVD-Hp in mouse brain was 92 pmol/g. By comparing the amounts of AEA and 2-AG to RVD-Hp, they found that the range levels of this peptide are similar to the levels of AEA and, thus, significantly lower than 2-AG (see above) (Buczynski and Parsons, 2010; Bauer et al., 2012). Using an immunoaffinity mass spectrometry technique to isolate and quantify peptides interacting with CB1 receptor, from mouse brain and mouse and human plasma samples, they found that these extended hemopressin peptides show a high-affinity binding to CB1 receptor. They also found that longer hemopressin-containing fragments (more than 17 amino acids) do not bind CB1 receptors and proposed that maybe these longer peptides are precursors to shorter ones that bind CB1 receptor (Bauer et al., 2012).

These recent identification of the first endogenous peptide modulators of cannabinoid receptors provide new functional characteristics and pharmacological properties to the already complex cannabinoid system. The new endogenous peptidergic regulation could be complementary to the classical lipid; however, there is a short of information on the interaction and relationship of these two components of the system compared to the classical ones. The study on the interaction between both classical and new cannabinoid molecules may shed light on a pharmacological use of these peptides to have not only a fine control of the cannabinoid system, but also to explore the modulation of it to aim different treatment outcomes.

### Hemopressin Peptides in Physiological Functions

The presence of the peptide cannabinoids in the brain and in other organs suggests a physiological role for them. Pharmacological manipulation has implied hemopressin peptide family activation of cannabinoid receptors in a diverse range of physiological functions. The first reports showed that independently of the route of administration, Hp has hypotensive (Rioli et al., 2003; Blais et al., 2005) and analgesic effects (Dale et al., 2005; Heimann et al., 2007). Interestingly, cannabimimetics neurological supra spinally mediate side-effects such as hypothermia, catalepsy and hypoactivity, which are responses typically associated with CB1 receptor agonists, were not reported after Hp administration (Heimann et al., 2007). While the major molecular targets of hemopressin peptides are the cannabinoid receptors, the mechanisms mediating the hypotensive effect are still unclear. However, different explanations have been suggested, such as vasodilation, release of nitric oxide (NO), ion channels activation or blockade, or even inhibition of endogenous peptidase activity leading to increased circulating levels of hypotensive peptides (Blais et al., 2005; Lippton et al., 2006; Macedonio et al., 2016). Recently, the effects of hemopressin extended peptide VD-Hp on blood pressure at the spinal level were analyzed. Low blood pressure induced by VD-Hp was not antagonized neither by the activation of CB1 receptor nor the inhibition of the NO synthase. Therefore, VD-Hp exerts its hypotensive effect via adrenoreceptor and not by CB1 or CB2 mechanisms nor systemic NO release (Li et al., 2014).

On the other hand, the analgesic effect of Hp is not conclusive, since Petrovszki et al. (2012) showed that intrathecally administered hemopressin could not decrease hyperalgesia at the spinal level in carrageenan-induced mechanical allodynic model. In agreement with Petrovzki results, Hama and Sagen (2011) showed that central or systemic administration of Hp did not have antinociceptive actions in a rat model of neuropathic spinal cord injury pain. However, it was recently observed that central and peripheral administration of VD-Hp had analgesic activity and the effects were completely blocked by CB1 antagonism (Han et al., 2014; Pan et al., 2014). Furthermore, central administration of higher doses of VD-Hp exerted hypoactivity and hypothermia and resulted in place-conditioned aversion besides food consumption behavior. In addition, in a tail-flick assay, rat VD-Hp exerted dose-dependent central antinociception through CB1 receptor, but not CB2 nor opioid receptors (Zheng et al., 2017). In a post-operatory pain model and phase I of formalin test, intracerebroventricular (i.c.v) administration of mouse VD-Hp induced dose-dependent analgesia in mice, which were markedly reduced by pre-treatment of CB1 antagonist. However, in the acetic acid-induced visceral pain model, supraspinal administration of mouse VD-Hp produced analgesic activities and the effects were significantly antagonized by both CB1 receptor antagonist AM4113 and TRPV1 receptor antagonist SB366791 (Zheng et al., 2018).

Furthermore, central effects of Hp on feeding behavior were investigated. Local and systemic administration of hemopressin caused a dose-dependent decrease of night-time food intake. Also, there were no medium-term adverse effects on feeding behavior such as nausea, aversion or sedation (Dodd et al., 2010; Dodd et al., 2013). In addition, Hp was able to overcome the orexigenic drive produced in leptin-deficient ob/ob mice (Di Marzo et al., 2001). However, the hypophagia was not produced by systemic administration of Hp in CB1^−/−^ mice, indicating this response to be mediated *in vivo* by CB1 receptors.

Evidence for the involvement of the ECS in neurological, psychiatric and neurodegenerative diseases has accumulated, providing leads for novel therapeutic approaches. In a mouse model of epilepsy (pilocarpine-induced seizures), the oral administration of Hp and NFKF, a shorter hemopressin peptide, showed a delay for the appearance of the first epilepsy symptoms and was proven to be more potent than Hp and cannabidiol to treat seizures. Also, a molecular docking study suggested that NFKF has a higher affinity binding to CB1 than cannabidiol and classical CB1 antagonists AM6538 and rimonabant. *In vivo* assays also showed that oral NFKL administration, a synthetic small peptide counterpart of human and mouse Hp, had similar anticonvulsant properties compared to NFKF (De Araujo et al., 2019).

Fogaça et al. (2015) showed an anxiogenic role for Hp (Fogaça et al., 2015). They observed that Hp induced anxiety in rodents not by CB1 receptors, but most likely via TRPV1 receptors. They proposed that the effect may be caused either by Hp binding directly to TRPV1 receptors as an agonist, or by increasing the eCB levels, such as AEA, in the same way as it increases eCB levels in the sensory peripheral neurons (Toniolo et al., 2014). On the other hand, RVD-Hp has distinct effects compared to Hp in mood disorders. While Hp was shown to induce anxiety- and depressive-like behaviors in rats, RVD-Hp treatment reduced these symptoms. Furthermore, this study showed that Hp increased monoamine oxidase (MAO-B) and catechol-O-methyltransferase (COMT) gene expression, the enzymes involved in the catabolism of catecholamines and serotonin, and decreased norepinephrine, dopamine and serotonin levels, downregulating the monoaminergic system, which may be a cause of behavioral disorders (Leone et al., 2017).

New research studies the effects of peptide cannabinoids on memory processes and diseases related to memory loss, such as Alzheimer's disease. The i. c.v infusion of Hp showed improved memory formation in novel object recognition (NOR) and object location recognition (OLR) tasks in healthy young mice, which were inhibited by RVD-Hp and VD-Hp. Interestingly, in a mouse model of Alzheimer’s disease, RVD-Hp and VD-Hp reversed memory impairment induced by the amyloid-β (1–42) (Aβ1-42), which were blocked by Hp and the CB1 antagonist AM251 (Zhang et al., 2017).

The detrimental effect of the excess of reactive oxygen and nitrogen species is well recognized in various types of diseases/pathologies including certain neurodegenerative (eg Alzheimer's disease) and inflammatory diseases. Increasing evidence also underscores the significance of endocannabinoid signaling in large number of pathological conditions, characterized by enhanced ROS production (Gallelli et al., 2018; Paloczi et al., 2018). Recently, an important cross-talk was reported between the ECS and various redox-dependent processes. Lipid eCBs may modulate oxidative stress and lipid peroxidation either by conveying beneficial free radical scavenging effects or through targeting cannabinoid receptors CB1 and CB2 or non-canonical cannabinoid receptors (Han et al., 2009; Mukhopadhyay et al., 2010; Hao et al., 2011). It was recently shown that peptide cannabinoids may modulate ROS and NO production in cultured hippocampal neurons. VD-Hp inhibited oxidative stress injury induced by Aβ1-42 by downregulating the intracellular lipid peroxidation product malondialdehyde and by upregulating the antioxidative enzymes catalase and glutathione peroxidase activities. Furthermore, VD-Hp prevented neuronal apoptosis via increasing Bcl-2 and decreasing Bax gene expression (Zhang et al., 2020a). In retinoic acid-differentiated human neuroblastoma SH-SY5Y cells treated with Aβ1-42, RVD-Hp prevented apoptosis increasing cell viability and proliferation, besides increasing neurite outgrowth and PSD-95 expression levels. In addition, RVD-Hp reversed Aβ1-42-induced Tau phosphorylation (Ser202) by decreasing the activity of PKA and increasing the deactivation of GSK-3β (Zhang et al., 2020b).

In an experimental autoimmune encephalomyelitis model of multiple sclerosis, the oral administration of rat hemopressin fragment NFKF improved clinical scores and locomotor activity. Interestingly, NFKF blocked the production of IL-1β and IL-6 in spleen leukocytes possibly via CB2 receptor activation, while in the inguinal lymph nodes only IL-1ß was significantly reduced, suggesting a role for these peptides in the treatment of multiple sclerosis (Heimann et al., 2020). However, effects within the CNS must be considered, since Xapelli et al. (2014) showed hemopressin increased oligodendrocyte differentiation of subventricular zone progenitor cells *in vitro* (Xapelli et al., 2014). Thus, hemopressin peptides may be of potential interest to treat demyelinating diseases.

Taken together, these findings reveal a potential therapeutic role for hemopressin peptides for treatment of psychiatric and neurodegenerative diseases. Due to its peptide nature, hemopressin peptides may provide a novel pharmacological tool that can be employed in therapeutic approaches. However, more studies are needed to address the mechanisms of action of hemopressin peptides.

### Anatomical Distribution and Biosynthesis of Peptide Endocannabinoids

Growing evidence suggests that peptide cannabinoids are in fact produced in the brain. However, there is still scarce data on the biosynthesis and cellular origin of hemopressin peptides in CNS and peripheral body tissues. The first evidence on the production of hemopressin peptides in the brain was obtained by Gelman and collaborators (2010) by using mass spectrometry analysis of blood and brain peptidomes. They compared the hemoglobin-derived peptides present in both blood and brain tissues, revealing that peptides found in the brain are largely distinct from those found in blood and heart. This result suggested that the brain hemopressin peptides are locally produced and not the result of blood or hemoglobin protein entries via the blood-brain barrier (Gelman et al., 2010). The discovery of these endogenous hemopressin peptides opened up a quest for their distribution in the nervous system. RVD-Hp was detected in several brain regions, such as hypothalamus, nucleus accumbens, olfactory bulb, cerebellum, prefrontal cortex and striatum, while VD-Hp was found in the olfactory bulb, hypothalamus and cerebellum (Gomes et al., 2009).

Recently, it has been shown that these peptides are present in specific areas of the CNS and body tissues (Hofer et al., 2015). Antibodies against the C-terminal of peptide cannabinoids were only found in noradrenergic neurons of the *locus coeruleus*, as well as in neurons of A1, A5 and A7 nuclei of the brainstem. In addition, axons positive for hemopressin peptides were found in projection regions of the *locus coeruleus,* including cerebral cortex, hippocampus, hypothalamus and spinal cord. The antibody was colocalized with tyrosine hydroxylase and galanin immunolabeling; however, it was not detected in dopaminergic neurons. As described above, there is evidence suggesting the modulatory effects of hemopressin peptides on monoaminergic signaling (Tanaka et al., 2014; Leone et al., 2017; Recinella et al., 2018).

In addition, hemopressin peptides were also present within the chromaffin cells of the adrenal medulla, which is the major site of noradrenaline biosynthesis in the body. Using a competitive enzyme-linked immunosorbent assay (cELISA) and a liquid chromatography–mass spectrometry (LC-MS/MS), it was observed that among hemopressin peptides, RVD-Hp had the highest expression on both brain and adrenal glands. However, the adrenal expression of hemopressin peptides was relatively higher compared to peptide levels obtained in the brain (Hofer et al., 2015).

**FIGURE 4:**
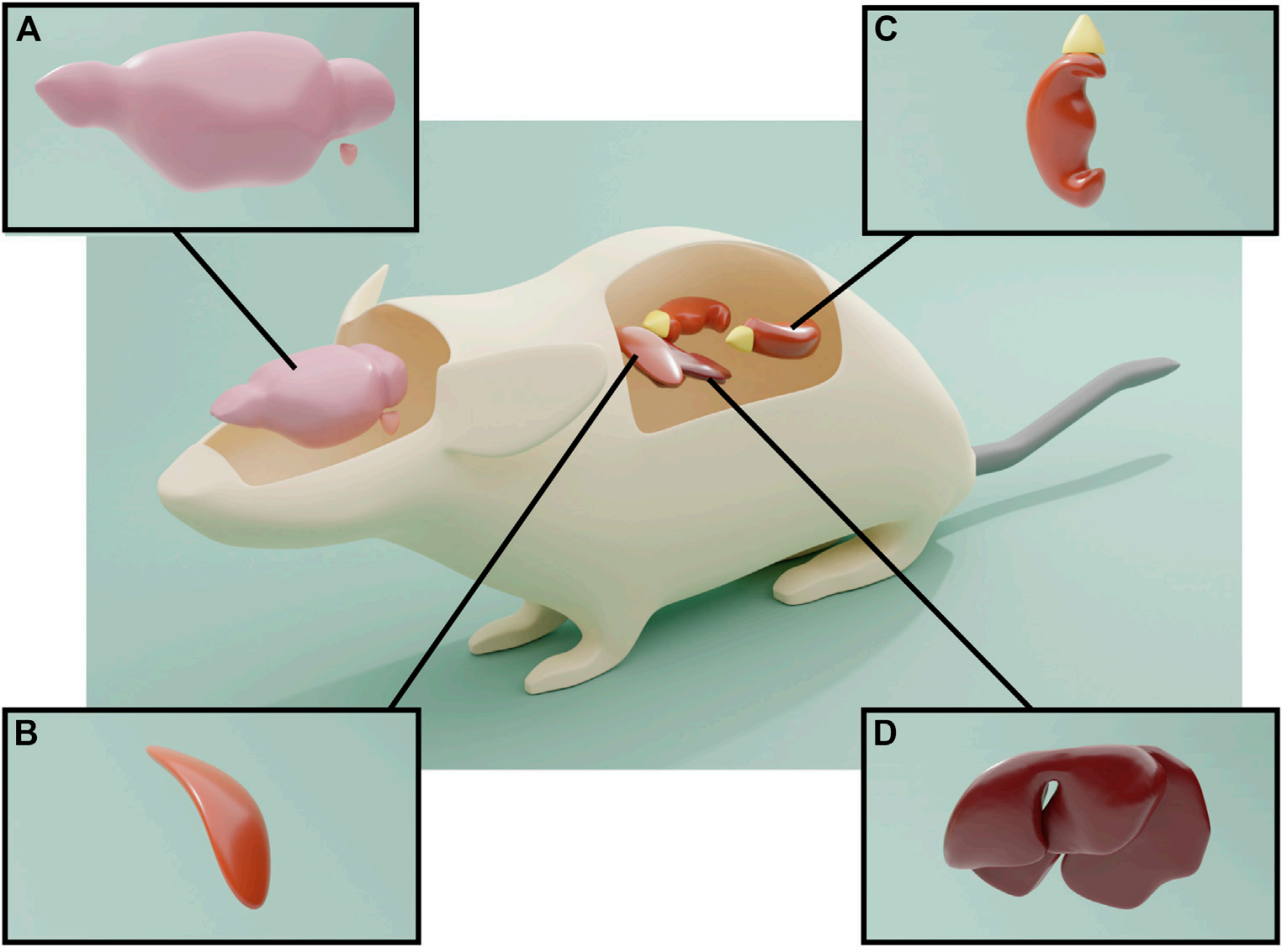
Expression of the hemopressin peptide. **(A)** Antibodies against the C-terminal of peptide cannabinoids only identified noradrenergic neurons of the locus coeruleus, as well as A1, A5 and A7 neurons in the brainstem. The antibody also identified axons in the throughout the brain, notably in the hippocampus and cerebral cortex and spinal cord, indicative of anterograde axonal transport of the hemopressin peptides. **(B)** Hemopressin peptides were also present within the chromaffin cells of the adrenal medulla. **(C)** and **(D)** Hemopressin peptides are also expressed in liver, spleen and kidney.

The presence of hemopressin peptides is not exclusive of the CNS and adrenal glands. RVD-Hp and hemopressin-23 (SALSDLHAHKLRVDPVNFKLLSH) are also expressed in the liver, spleen and kidney (Petrucci et al., 2017) (Figure 4). Interestingly, the RVD-Hp levels in the brain are much lower in comparison with these organs; the highest levels of expression were observed in the adrenal glands, liver and spleen, followed by kidney. The brain levels of hemopressin-23 are about three times higher than those of RVD-Hp, and because of that, it was proposed that hemopressin-23 could be a precursor peptide for RVD-Hp. Petrucci et al. (2017) proposed that hemopressin-23 could be the peptide detected in *locus coeruleus* projection fibers by the C-terminal antibody prospection performed by Hofer et al. (2015). In turn, it was proposed that the adrenal gland could be the major source of hemopressin peptides in the body due to its high levels and because adrenalectomy decreases RVD-Hp in liver, kidney, spleen and brain (Petrucci et al., 2017). Conversely, in the same study, the production of hemopressin peptides was analyzed in pathological conditions and was shown to be increased in liver, kidney and adrenals upon an inflammatory stimulus. Nevertheless, it was observed that after ischemia reperfusion injury, the levels of RVD-Hp were only increased in liver and kidney, suggesting that, in response to tissue damage, their production of RVD-Hp may be independent of the adrenal gland.

Although new data on the role of hemopressin peptides are produced constantly, there is still no conclusive evidence on their biosynthetic pathways. Intracellular peptides are commonly produced by limited proteolysis from a specific set of intracellular proteins (De Araujo et al., 2019); however, the biosynthetic pathway that leads to hemopressin peptides is not yet understood. Interestingly, since Hp was observed to be a substrate for the widely distributed endopeptidases 24.15, 24.16, and ACE, which also catalyze other bioactive peptides such as bradykinin, angiotensin I and some opioid peptides (Rioli et al., 2003), it may be suggested that these endopeptidases are also involved in the biosynthetic pathways of hemopressin peptides. However, to date, there is no direct evidence of the degradation of α-hemoglobin and β-hemoglobin by these enzymes to form hemopressin peptides.

There is still no direct evidence supporting the hypothesis that hemopressin production is related to hemoglobin degradation and that the production of these peptides is an independent event altogether. However, surprisingly, the α- and β-chains of hemoglobin were detected in brain cells as well as in their messenger ribonucleic acid (mRNA). They were found in dopaminergic cells of the *substantia nigra* and midbrain, as well as in GABAergic cells of striatum and pyramidal cells of the cerebral cortex (Biagioli et al., 2009). Both mRNA expression and the immunodetection of hemoglobin chains were performed in cultures, showing positive results for neurons, astrocytes, oligodendrocytes and differentiated human neuroblastoma cells (Biagioli et al., 2009; Richter et al., 2009; Gelman et al., 2010).

Furthermore, nothing is known about possible secretory pathways for these peptides. Because there is a significant portion of intracellular peptides that can be secreted and bind to cell surface receptors, it is possible that a secretion mechanism exists for hemopressin peptides (De Araujo et al., 2019). Due to the intracellular presence of hemopressin peptides in at least some populations of neurons (Hofer et al., 2015), it is possible that they coexist with lipid endocannabinoids and that there is an interaction of these two components (peptides and lipids). In addition, the intracellular presence of these peptides suggests the regulation of intracellular cannabinoid receptors. It is possible that they may activate mitochondrial and endosomal/lysosomal cannabinoid receptors directly or by co-modulation with lipid endocannabinoids.

In summary there are few studies based on the biosynthesis and production of hemopressin peptides, and many alternative hypotheses have been proposed, such as proteosomal or other intracellular proteases cleavage (calpains, caspases, and cathepsins) (Gelman and Fricker, 2010). In addition, it was suggested that the biosynthesis of hemopressin peptides may be through *de novo* expression or fast differential processing of the hemoglobin alpha 1/2 (HBA1/HBA2) gene products (Petrucci et al., 2017). Nevertheless, the presence of hemopressin peptides in the brain strongly points to their local production and the regulation of their expression in pathological events suggests their important physiological role.

### Modulation of Cannabinoid Receptors by Peptide Cannabinoids

**FIGURE 5:**
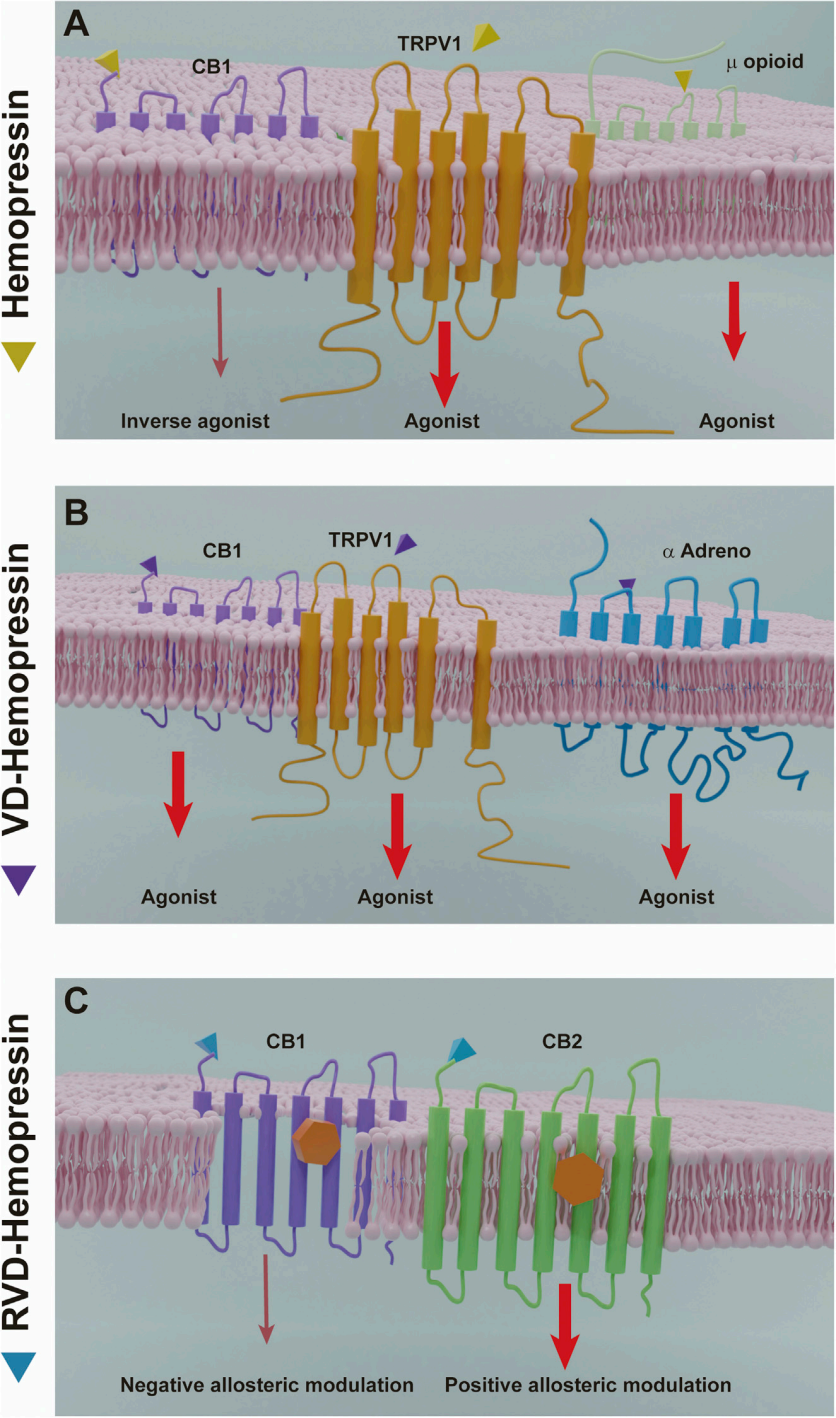
Hemopressin peptides mechanisms of action. **(A)** Hemopressin acts as inverse agonist of CB1 and as an agonist of the receptors TRPV1 and the µ opioid receptor. **(B)** VD-hemopressin is an agonist of the CB1 receptor and for control nociception and blood pressure can interact with the TRPV1 NFF and α adrenergic receptor. **(C)** RVD-hemopressin can act as a negative allosteric modulator of CB1 and a positive allosteric modulator of CB2. Thinner arrows (dark red) indicate inverse agonist or negative allosteric effects by hemopressins. Larger arrows (light red) indicate agonist or positive allosteric modulation.

Initially, Hp was characterized as a selective ligand for CB1 with inverse agonistic properties (Heimann et al., 2007). Besides, there is evidence that Hp could interact with the endogenous opioid system and its receptors and with other non-canonical cannabinoid receptors such as TRPV1 (Figure 5). In addition, it was reported that VD-Hp and RVD-Hp have a CB1 agonist activity in contrast to Hp. RVD-Hp and VD-Hp were found to exhibit a high-affinity binding to CB1 and a lower affinity to CB2. These peptides bind cannabinoid receptors in nanomolar concentrations. While binding, they can displace cannabinoid agonists such as CP55,940; however, this efficiency is lower if compared to rimonabant. It was shown that these peptides exhibit functional selectivity at the CB1 receptor; nevertheless, they may activate signaling pathways distinct from classical CB1 receptor agonists (Gomes et al., 2009).

A comparison of the time course of ERK phosphorylation by the peptide ligand with that of the classical CB1 ligand, HU-210, showed differences in temporal dynamics. The extended N-terminal hemopressin peptides exhibited a peak activity at 30 min compared to HU-210, which is at 5 min. Moreover, the increase in the levels and in the rate of ERK phosphorylation induced by these peptides is lower than those induced by HU-210. In contrast, the increase in intracellular Ca^2+^ levels produced by these peptides is much more robust than the HU-210-induced increase, and the effects of the hemopressin peptides on signaling is only partially blocked by pertussis toxin, in contrast to HU-210, which is completely blocked indicating differential G_i_ protein mediation (Gomes et al., 2009). Furthermore, the peptides produced similar responses compared with HU-210 in a series of assays measuring functional consequences of receptor activation, including neuronal outgrowth in neuro 2A cells and internalization of myc-tagged CB1 receptors expressed in CHO cells. It was also observed that Hp modulated ACEA upregulation of CB1 expression. In a model of primary cultured hippocampal neurons and in mouse hippocampus treated with AEA there was an upregulation of the expression of CB1 receptor, which was abolished by Hp. Interestingly, this effect was observed only in cell membrane CB1 population and not in mitochondrial CB1, confirming that hemopressin is a cell-impermeant CB1 antagonist (Ma et al., 2016; Ma et al., 2015). In addition, there is evidence that VD-Hp interacts with other receptors besides CB1 to control a variety of physiological functions such as nociception and blood pressure by activating both TRPV1 neuropeptide FF receptor and α-adrenoreceptor. However, the signaling pathways involved in these processes are still unknown.

Nonetheless, it was recently proposed that the N-terminal extended hemopressin peptides have a negative allosteric modulation for CB1 receptor and not an agonist activation (Figure 5). Using a radioligand displacement assay, it was shown that the classical CB1 agonists [^3^H]CP55,940 and [^3^H]WIN55,212–2 are partially displaced by RVD-Hp. Additionally, a dissociation kinetic study showed that [^3^H]CP55,940 in the presence of RVD-Hp led to an increase in the dissociation rate constants. Correlating these data with the ternary complex model, which describes the interaction between an orthosteric ligand and an allosteric modulator, it was revealed that RVD-Hp had a negative allosteric modulation on the CB1 receptor (Bauer et al., 2012). To investigate the effect of RVD-Hp on the CB1 activation and cAMP accumulation, RVD-Hp was compared with CB1 agonists and antagonists. Surprisingly, it was observed that CB1 agonist stimulation increased the basal cAMP and an inverse agonist stimulation decreased its accumulation, suggesting a CB1/G_s_ mediated pathway. However, RVD-Hp did not elicit any changes in basal cAMP levels by itself, and a reduction was only observed in the presence of a CB1 agonist (WIN55,212-2 and 2-AG), indicating an allosteric modulation. In addition, RVD-Hp was able to induce CB1 receptor internalization (Bauer et al., 20126).

Following this idea, the negative allosteric modulation of RVD-Hp on synaptic transmission was tested by using an autoptic model of hippocampal neurons, which enables to test cannabinoid signaling modulation of DSE, a form of retrograde inhibition involving eCBs and CB1 receptors. It was shown that RVD-Hp attenuated DSE and did not directly inhibit CB1 receptors, while Hp did not inhibit DSE (Straiker et al., 2015). Moreover, it was observed that ‘strong’ DSI induced by a 5 s depolarization of CA1 pyramidal neurons was fully blocked by AM251, a lipophilic and permeable CB1 antagonist, but only partially and reversibly reduced by Hp, a non-permeable inverse agonist. This difference is due to the participation of the mitochondrial CB1 receptor in the modulation of DSI. Also, Hp fully blocked the depression of evoked inhibitory post-synaptic currents (IPSCs) elicited by HU210 (Bénard et al., 2012).

On the other hand, it was recently reported that RVD-Hp exerts a positive allosteric modulation role on CB2 receptors in contrast to its effects on CB1 receptors (Petrucci et al., 2017). Using a competitive radioligand binding assay in CB2 receptor-transfected membranes, it was shown that RVD-Hp is not able to displace [^3^H]CP55,940 nor [^3^H]WIN55,212–2, but favors their binding in both cases. The dissociation kinetics of [^3^H]CP55,940 was also measured from CB2 receptors and it was observed that RVD-Hp induced a reduction on the dissociation ratio. Furthermore, it was found that this peptide did not affect the internalization of CB2 receptors via β-arrestin2 (Petrucci et al., 2017). The evaluation of functional properties of RVD-Hp showed that the co-activation of the orthosteric site of CB2 is necessary for triggering or inhibiting CB2 receptor-mediated signaling pathways. Also, when RVD-Hp was incubated in presence of CP55,940 or 2-AG, it induced a significant potentiation of cAMP inhibition via CB2-mediated G-protein (G_αi_). Intriguingly, RVD-Hp exerted an even stronger potentiation of 2-AG-mediated cAMP inhibition compared to CP55,940. However, RVD-Hp did not influence CP55,940-induced β-arrestin2 recruitment. Recently, there are other peptide endocannabinoids suggested to have an allosteric modulation of CB1/CB2 receptors such as NFKF (Heimann et al., 2020).

The difference between Hp activation and the N-terminal extended peptides suggests that they have qualitatively different modes of binding to cannabinoid receptors; however, the characterization of the binding sites is not yet fully understood. The fact that hemopressin peptides can bind to a receptor and activate it in a different manner may be due to their “selective functionality”. This “functional selectivity” can activate or inhibit characteristic patterns of downstream effector pathways depending on their structures and the conformational changes induced in the receptor resulting in ligand-dependent receptor signaling (Kenanin, 2007). However, more studies are needed to elucidate the mechanisms by which hemopressin peptides modulate cannabinoid receptors.

The first evidence on allosteric modulation of cannabinoid receptors was described with synthetic molecules, however, there are already a few endogenous allosteric modulators characterized so far. Despite the fact that endogenous molecules that were described as allosteric modulators of CB1 are all of lipid nature, the recent identification of hemopressin peptides as endogenous allosteric modulators of the cannabinoid receptors has provided new evidence of novel mechanisms of receptor modulation. Therefore, the fact that N-terminal extended hemopressin peptides have allosteric modulation for CB1 and CB2 provides opportunities for manipulation of selective signaling pathways in a spatially and temporally restricted manner to modulate and develop therapeutic approaches that are subtype-specific and, in some cases, pathway-specific. Hemopressin peptides allosteric modulation may have important physiological and endogenous regulatory consequences able to enhance or limit the receptor activity providing a fine-tuning mechanism of downstream pathways and also allow for improved spatiotemporal regulation of an endogenous ligand. Furthermore, these molecules can provide new opportunities to intervene in the physiology of CB and non-canonical CB receptors in pathological conditions. However, most studies on the allosteric modulation of hemopressin peptides were carried out on isolated membranes or *in vitro* models that overexpressed receptors, so it would be necessary to carry out experiments on cellular or *in vivo* models.

The fact that RVD-Hp was proposed to be a CB2 positive allosteric modulator is interesting because, for the first time, the existence of an allosteric modulator for CB2 receptor was considered. On the other hand, new ways of cannabinoid receptor modulation have been proposed, such as receptor internalization and desensitization. Cannabinoid agonists can also signal differentially via β-arrestins coupled to mitogen-activated protein kinases, subsequently promoting different degrees of receptor internalization and agonist desensitization. Although hemopressin peptides are known to cause receptor internalization, the mechanisms involved on it are still unknown.

Hemopressin peptides were shown to regulate the function of other non-canonical cannabinoid receptors in a diverse of physiological functions (Li et al., 2014; Sampaio et al., 2015, Fogaça et al., 2015; Szlaviz et al., 2015; Pan et al., 2015; Zheng et al., 2018; Leone et al., 2018) (Table 2). In an animal model of anxiety, the i. c.v administration of Hp induced anxiogenic-like effects that were blocked by the addition of a TRPV1 antagonist (Fogaça et al., 2015;). In addition, a rapid effect of Hp on (Na^2+^K^+^)-ATPase of LLC-PK1 cells may be dependent on the TRPV1 receptor since it was blocked by a TRPV1 antagonist (Sampaio et al., 2015). Also, the incubation with a TRPV1 antagonist blocked the analgesic effect of VD-Hp in the acetic acid-induced visceral pain model (Zheng et al., 2018). Furthermore, there is evidence that Hp has a modulatory effect on the opioid system. Hp enhances the affinity for *µ* receptors and increases G protein activation (Szlaviz et al., 2015). In addition, it was observed the involvement of calcium-activated K^+^ channels on Hp-induced analgesic effect on a chronic constriction injury-induced hyperalgesia model (Scrima et al., 2010).

**TABLE 2:** Hemopressin peptides-activated signaling pathways.

Peptide	Receptor	Physiological effects	Receptor signaling	References
Hp	CB1	Antinociceptive effects	Decreased G protein activation	Heimann et al., (2007)
			Increased adenylyl cyclase activity	
			Decreased pERK signaling	
Hp	CB1	Anorexic effects	Inhibited agonist-induced CB1 internalization	Dodd et al. (2010)
Hp	Calcium-activated K^+^ channel	Analgesic effects	Decrease KCl-induced calcium flux	Toniolo et al. (2014)
			Blocked the channel reversed Hp-induced antinociception	
Hp	CB1/µ opioid receptor	Not shown	Increased G protein activation	Szlavicz et al., (2015)
Hp	Not shown	Not shown	Weakly increased G protein activation	Dvoracsko et al. (2016)
Hp	CB1/TRPV	Modulation of Na + transport	Increased na^+^/K^+^–ATPase activity via TRPV1	Sampaio et al. (2015)
			Decreased na+/K^+^–ATPase activity via CB1	
			Increased AMPc levels via CB1	
			Decreased pERK via CB1	
Hp	TRPV	Anxiogenic-like effects	Not shown	Fogaça et al. (2015)
Hp	Not shown	Anxiogenic-like effects	Decreased monoamine activity	Leone et al. (2017)
			Increased expression of monoamine oxidase and catechol-O-methyltransferase gene	
VD-Hp/ RVD-Hp	CB1	Neurite outgrowth	Increased intracellular Ca^2+^ levels	Gomes et al. (2009)
			Induced CB1 internalization	
			Increased pERK levels and rate but lower compared to classical cannabinoids	
			No effect on G protein activation	
			Induced faster and robust increase in Ca^2+^ release compared to classical cannabinoids	
VD-Hp	Neuropeptide FF receptors	Analgesic effects	Not shown	Pan et al. (2015)
VD-Hp	TRPV1	Analgesic effects	Not shown	Zheng et al. (2018)
VD-Hp	α-adrenoreceptor	Hypotensive effect	Not shown	Li et al. (2014)
RVD-Hp	CB1 negative allosteric modulation	Not shown	Decreased AMPc levels (co incubation with CB1 agonist)	Bauer et al. (2012)
			Decreased G protein activation (co incubation with CB1 agonist)	
			Induced CB1 internalization	
RVD-Hp	CB1 negative allosteric modulation	Attenuated 2-AG mediated DSE	Reduce EPSC	Straiker et al. (2015)
RVD-Hp	CB2 positive allosteric modulation	Not shown	Decreased AMPc levels (co-incubation with classical agonist)	Petrucci et al. (2017)
			Increased G protein activation (co-incubation with a classical agonist)	
RVD-Hp	Not shown	Anxiolytic effects	Increased monoamine activity	Leone et al. (2017)
			Decreased expression of monoamine oxidase and catechol-O-methyltransferase gene	
RVD-Hp	Not shown	Anorexic effects	Increased intracellular Ca^2+^	Leone et al. (2018)
			Inhibited FAAH gene expression	

There is also evidence showing that hemopressin peptides can interact with other endogenous systems. Pan et al. (2015) showed that VD-Hp modulated the pharmacological action of neuropeptide FF supporting the hypothesis that hemopressin peptides can directly or indirectly interact with other G-protein coupled receptors. It was also reported that RVD-Hp co-localizes with the neuropeptide galanin, which is involved in the modulation and inhibition of action potentials in neurons, suggesting that RVD-Hp modulates other neuropeptide functions (Hofer et al., 2015). Furthermore, it was shown that RVD-Hp induced anxiolytic effects and decreased the expression of MAO-B and COMT gene expression (Leone et al., 2017). Moreover, it was proposed that hemopressin peptides may modulate the enzymes of endocannabinoid degradation resulting in increased endocannabinoid levels. It was observed that RVD-Hp decreases FAAH gene expression in the hypothalamus (Leone et al., 2018). Also, a FAAH inhibitor potentiated the antinociception induced by Hp, reinforcing the interplay between the ECS enzymes and hemopressin peptides (Toniolo et al., 2014) (Table 2).

These evidences indicate that not all effects of hemopressin peptides seem to depend on CB-mediated mechanisms. It was proposed that these peptides can directly or indirectly modulate the functions of non-canonical cannabinoid receptors and other endogenous systems in the regulation of diverse physiological functions. In addition, it has been suggested that the regulatory mechanism is not evoked by hemopressin cannabinoids agonists/antagonists by themselves, but by the modulation of cannabimimetic actions by other cannabinoids. In this manner, the hemopressin peptides could upregulate the endocannabinoid production and the subsequent endocannabinoid release. These alternatives increase the availability of eCBs, interestingly because they can lead to an amplification of endocannabinoid tone ensuring the enhancement of specific pathways without altering other inactive pathways. Thus, expanding research in cannabinoid signaling in its subcellular, cellular and regional levels may yield a plethora of possibilities unexplored in medicine.

## Conclusion

Due to the advances on the endocannabinoid system studies, it is becoming clear that its role is not restricted to the brain, but also to a variety of body processes in order to maintain homeostasis. The presence of these hemoglobin-derived peptide cannabinoids in the brain and in other organs of the body suggests that they are new endogenous CB-receptor ligands. Thus, they not only regulate different physiological functions but also coexist and interact with lipid endocannabinoids.

The nature of hemopressin peptides, as hydrophilic molecules, provides new biological properties on cannabinoid signaling. The various lengths among peptide cannabinoids allow the selection of different modes of cannabinoid receptor binding, thereby stimulating distinct intracellular signaling pathways. Because peptide endocannabinoids allosterically bind to CB receptors, they provide a new way to modulate receptor responses in a spatially and temporally restricted manner.

For the application of hemopressin peptides for therapeutical purposes several issues will have to be addressed, for example, metabolic stability *in vivo*, self-assembly, fibril structure formation, high molecular weight, peptide nature and low penetration through blood-brain barriers. The low penetration through BBB of some hemopressin peptides may provide peripherally restricted agonists and antagonists for cannabinoid receptors avoiding central effects. Another application involves the inability of hemopressin peptides to traverse cell membranes, which permits the discrimination between cell membrane and intracellular cannabinoid receptor population. Also, the potential of peptide endocannabinoids to allosterically activate distinct intracellular signaling pathways compared to classical cannabinoid receptor ligands may reveal an application of these peptides in fine-tuning different therapeutical purposes. One of the possibilities is the usage of cannabinoid mixtures - lipids and peptides - that could present synergistic beneficial effects. These exclusive properties of peptide cannabinoids may be of great relevance to be explored in medicine yielding a plethora of future pharmacological applications.

## Author Contributions

All authors listed have made a substantial, direct, and intellectual contribution to the work and approved it for publication.

## Funding

This research was funded by INCT/CNPq, Grant Number 465489/2014-1 and FAPERJ, Grant Number E-11/2018E_11/2018.

## Conflict of Interest

The authors declare that the research was conducted in the absence of any commercial or financial relationships that could be construed as a potential conflict of interest.
